# Predictive effect of nonarteritic anterior ischemic optic neuropathy on the development of cerebral small vessel disease: a retrospective study

**DOI:** 10.3389/fneur.2024.1400452

**Published:** 2024-11-15

**Authors:** Xu Han, Hongyang Li, Zhaoyang Meng, Yanling Wang

**Affiliations:** Department of Ophthalmology, Beijing Friendship Hospital, Capital Medical University, Beijing, China

**Keywords:** nonarteritic anterior ischemic optic neuropathy, cerebral small vessel diseases, magnetic resonance imaging, white matter hyperintensities, retrospective study

## Abstract

**Objective:**

The study aims to determine the correlation between cerebral small vessel diseases (SVDs) and nonarteritic anterior ischemic optic neuropathy (NAION). Additionally, investigate whether NAION raises the risk of an increased total cerebral small vessel disease score (CSVD score) compared to control group without ocular conditions.

**Methods:**

101 controls without any retinal illness and 61 individuals with NAION were enrolled for this retrospective case control study. Ophthalmic examinations and brain magnetic resonance imaging (MRI) scans were performed on all participants. Data on demographics and clinical characteristics were obtained from hospital medical records. We evaluated and compared the distribution of SVDs and rated the total CSVD score based on SVD indications observed on MRI scans.

**Results:**

SVDs were more frequently in NAION individuals than in control group (82%, *p* < 0.001), and their odds ratio was 4.11 (95%CI: 1.93–8.79, *p* < 0.001). The ordinal logistic regression showed patients in NAION group had 3.08-, 5.66- and 2.90-times higher risk than in control group, at each point of the white matter hyperintensity (WMH) score (95%CI: 1.43–6.79, *p* = 0.003), perivascular spaces (PVS) score (95%CI: 2.31–14.9, *p* < 0.001) and CSVD score (95%CI: 1.32–6.51, *p* = 0.005) respectively. Dyslipidemia presented a higher risk in the presence of SVDs (*p* = 0.008, OR = 2.31, 95%CI: 1.20–4.44) and WHM score (*p* = 0.018, OR = 2.22, 95%CI: 1.07–4.70). There was no significant difference between NAION group and controls in sex, age, or other past medical characteristics.

**Conclusion:**

The predictive effect of NAION on SVDs is possible as NAION patients have an increased risk with SVDs. Brain MRI scans and the control of risk factors associated with SVDs should be recommended for individuals who develop NAION.

## 1 Introduction

In elderly individuals, nonarteritic anterior ischemic optic neuropathy (NAION) is a prominent cause of visual impairment accompanied by optic nerve edema (1). It is a complicated condition thought to damage circulation of short posterior ciliary arteries caused by various systemic and ocular predisposing risk factors (2, 3). Several studies have shown the most common associated risk factors, including diabetes mellitus, hypertension, hyperlipidemia, atherosclerosis and obstructive sleep apnea syndrome, which also increase the risk of coronary heart disease or cerebral ischemic infarction. However, owing to overlapping risk factors, it is challenging to ascertain if NAION is an independent risk factor for cerebral vascular diseases. Therefore, the association of NAION and cerebral vascular diseases is controversial.

Cerebral small vessel diseases (SVDs) refer to a class of age-related degenerative alterations that are thought to be caused by the disease breaking cerebral small arteries, arterioles, capillaries, and small veins (4). There are a number of diagnosed structures described in neuroimaging: lacunar infarction, white matter hyperintensity(WMH), perivascular spaces (PVS), and cerebral microbleed (CMB). SVDs causes physical disorders and cognitive impairment secondary to stroke and dementia (5), particularly in individuals with more severe brain parenchymal injuries (6, 7). To prevent dementia and stroke, it is crucial to identify predictive factors of SVDs. Since NAION is an optic nerve disease that can affect small vessels, recent studies have focused more attention on the correlation between NAION and SVDs. Previous studies have reported brain magnetic resonance imaging (MRI) scans of patients with NAION have a significantly higher frequency of WMH (8). Kim MS et al. reported the relationship between NAION and each subtype of SVDs (9). However, there are little study has quantitatively investigated correlation between NAION and the level of severity of total SVDs. To evaluate the degree of SVDs, the developed “total CSVD score” based on MRI has been proposed. Therefore, this visual scorning systems make it possible to analyze the correlation of NAION and overall SVDs burden.

Hence, our research aims to determine the relationship between NAION and various imaging phenotypes of SVDs, presenting evidence in support of the hypothesis that NAION predicts the development of SVDs. To do this, we firstly assessed the distribution of SVDs in patients with NAION and control group. Subsequently, we evaluate the relationship between NAION and SVDs severity from the perspective of total CSVD score.

## 2 Methods

### 2.1 Design and participants

We retrospectively collected original records of patients with NAION and the control group who visited Capital Medical University Beijing Friendship Hospital from June 2019 and Oct 2022. This single-center, retrospective, case control study received approval from the Beijing Friendship Hospital ethics committees, and written informed consent was acquired from each individual prior to participation.

**Figure 1:**
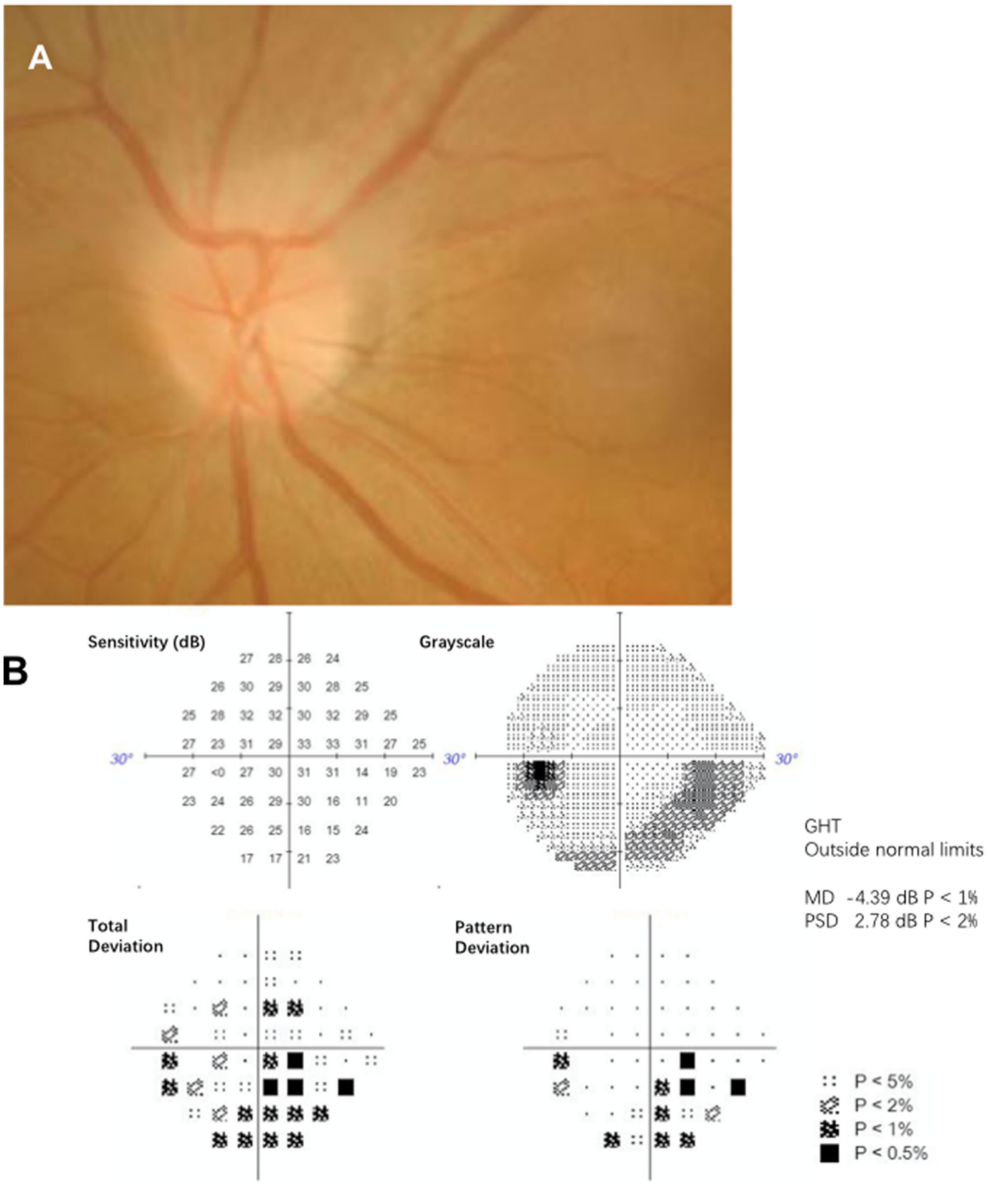
(A) Funduscopic examination from one NAION participant (age 61) shows segmental swelling at upper edge of optic disc. (B) This participant shows an inferior altitudinal defect on visual field corresponding to optic disc edema.

Two neuro-ophthalmologist (HX and LHY) confirmed the inclusion criteria for NAION subjects were as follow (10): [1] acute painless loss of visual acuity; [2] Automatic perimetry examination revealed partial visual field defect; [3] funduscopic examination shows diffuse or segmental optic disc edema (Figure 1); and [4] exclude other optic nerve diseases such as glaucoma, optic neuritis and arteritic anterior ischemic optic neuropathy. The following were the criteria for exclusion: [1] bilateral optic nerve involved at initial presentation; [2] other retinal diseases affecting visual acuity or visual field; and [3] severe medical condition, such as autoimmune disease, serious heart disease or cancer.

**Figure 2:**
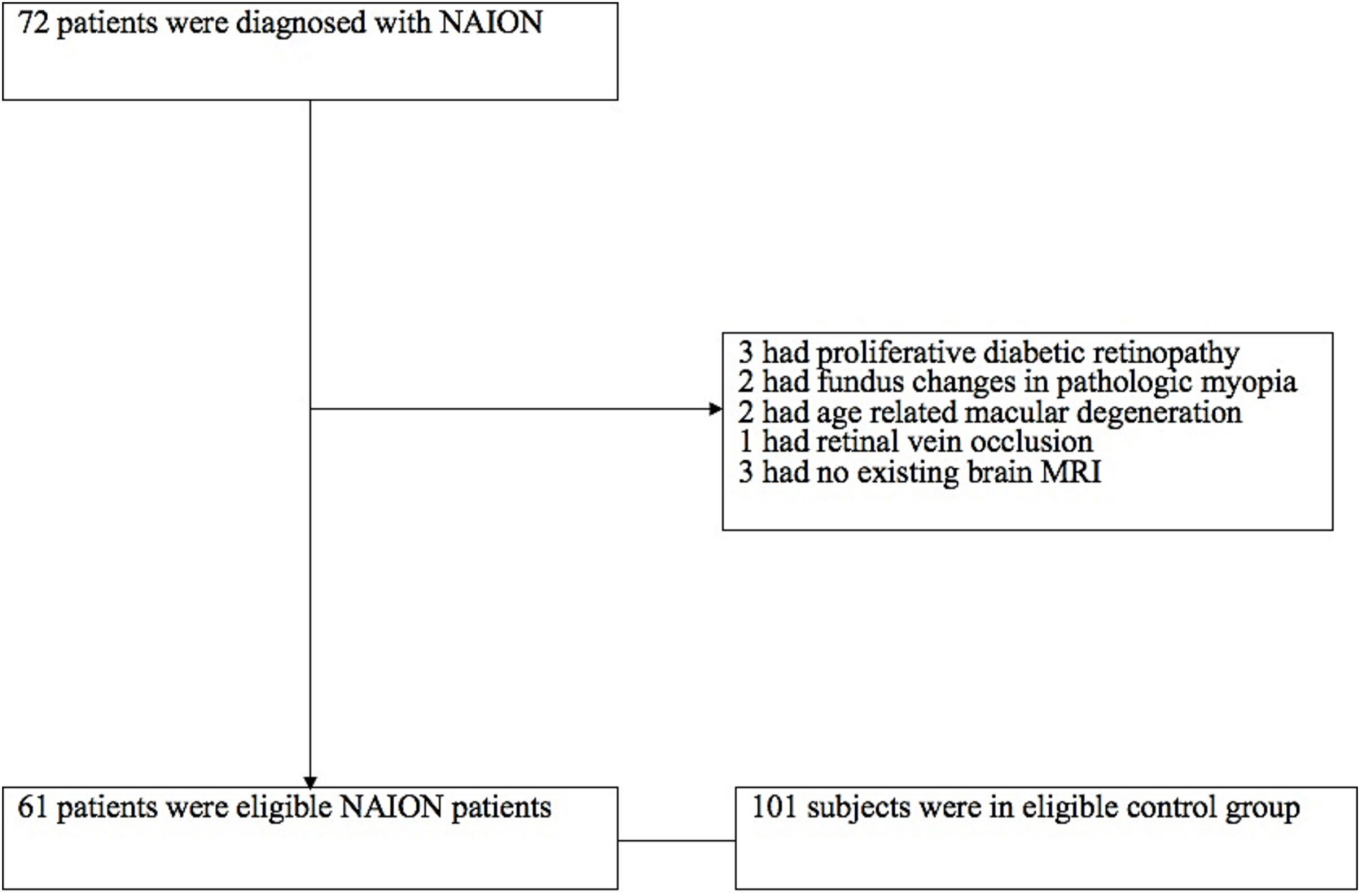
The inclusion and exclusion procedures used in this study. NAION was identified in a total of 72 patients. The reasons for exclusion are displayed on the right side. Finally, 61 individuals with NAION were collected in our analysis.

Participants in control group were enrolled by retrospectively reviewing the medical records who visited our department between June 2019 and Oct 2022. We identified the control group who had no any abnormal signs in fundus photography examination including optic disc, macula, retinal vessels and other fundus disease. Participants who were unable to cooperate with the MRI scans were excluded. Figure 2 illustrates the procedure for inclusion and exclusion.

### 2.2 Medical histories and clinical evaluation

All subjects underwent a standardized ophthalmic evaluation consisting of best-corrected visual acuity, visual field (VF), slit-lamp microscope, fundus photography, and optical coherence tomography scans. The VF was evaluated using the Humphrey Field Analyzer (Zeiss Humphrey Field Analyzer HFA 745-I, Germany) and mean deviation (MD) of the central 30–2 program was used to quantitatively assess the VF defects. The field of 30°fundus photograph of optic disc and macula was collected using a digital retinal camera (Zeiss Clarus 500, Germany).

We retrospectively collected demographic information and medical history on carotid artery stenosis, cardiovascular disease, hypertension, diabetes mellitus, dyslipidemia, stroke or transient ischemic attack (TIA), history of smoking, alcohol use and obesity through hospital medical record system to assess vascular risk factors.

### 2.3 Brain MRI acquisition and analysis

**Figure 3:**
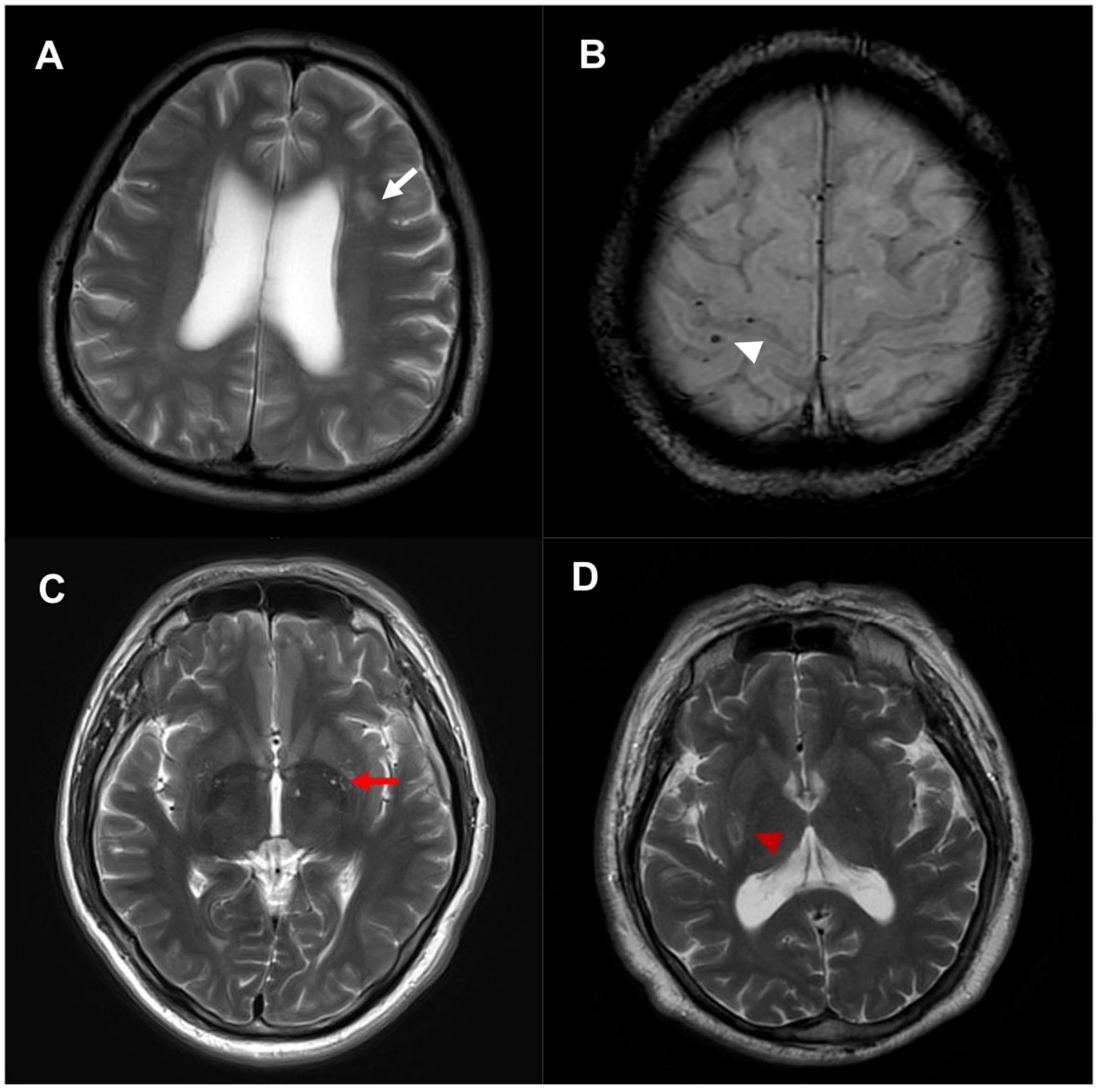
Cerebral small vessel diseases in brain MRI images: (A) White matter hyperintensity (white arrow) in T2-weighted image. (B) Cerebral microbleed (white arrowhead) in SWI image. (C) Perivascular spaces (red arrow) in T2-weighted image. (D) Lacunar infarction (red arrowhead) in T2-weighted image. MRI: magnetic resonance imaging; SWI: susceptibility-weighted imaging.

Participants received brain MRI scans at 3 Tesla using a GE Discovery MR 750 machine from GE Healthcare in Waukesha, USA. The images obtained included T1-weighted, T2-weighted, fluid-attenuated inversion recovery-weighted (FLAIR), diffusion-weighted imaging (DWI), and susceptibility-weighted imaging (SWI). SVDs is consisted of lacunar infarctions, WMH, CMB, and enlarged perivascular space (PVS). We conducted all MRI visual assessments of SVDs with reference to STRIVE guidelines: [1] lacunar infarction was defined as a circular or oval-shaped cavity and central hypointense signal with a surrounding hyperintense rim on FLAIR images. [2] WMH was defined as vascular lesions of white matter with hyperintense signals on T2-weighted sequences while appear as hypointensities on T1-weighted sequences. [3] CMB was defined as a small round hypointense lesion that can appear on SWI sequences with a general diameter of 2–5 mm. [4] PVS was described as fluid-filled areas with a signal intensity comparable to cerebrospinal fluid on each image that following the course of blood vessels (11). Figure 3 illustrates SVDs in brain MRI images. Total WMH lesions score was calculated on periventricular WMH and deep WMH Fazekas grade scale (with the maximum possible score being 6): Periventricular WMH was graded as 0 points: absence, 1 point: “caps” or “pencil-thin lining,” 2 points: smooth “halo,” 3 points: irregular lesions extends into the deep white matter. Deep WMH was graded as 0 points: absence, 1 points: “punctate foci,” 2 points: beginning confluence of foci, 3 points: large confluent areas (12). Total PVS score was rated with a qualitative rating scale: the number of lesions in both the basal ganglia and centrum semiovale regions were rated 0 (none), 1 (1–10), 2 (11–20), 3 (21–40), and 4 (>40) (13). Total CSVD score was computed using a four points scale proposed by previously studies (14). One point was awarded to total CSVD score by counting the presence of each of the following: lacunar infarction ≥1, CMB ≥ 1, PVS ≥ 10 in the basal ganglia, and periventricular WMH score was 3 or deep WMH score ≥ 2. Two independent trained clinicians assessed the presence of SVDs on MRI. Each participant’s total CSVD score could be determined when the two independent clinicians’ score were the same. When disagreements arose, the two clinicians discussed to reach a consensus outcome.

### 2.4 Statistical analyses

The R statistical analysis package was used for statistical analysis. The Shapiro–Wilk test was applied to evaluate the normal distribution. The description of normal continuous variables was given as means ± standard deviations. The differences in normal continuous variables between NAION group and control group were compared using the *t*-test. To compare nonparametric data, the Wilcoxon rank-sum test was used. Absolute numbers (relative frequencies) were used to represent categorical data. The categorical data between the two groups was compared using the χ2 test and the Fisher’s exact test. Multivariate logistic regression and ordinal logistic regression were used to evaluate the relationship between NAION with other risk variables and the overall burden of CSVD. The 95% confidence intervals (CI) for the odds ratio (OR) were computed. The statistically significant level was *p* < 0.05.

## 3 Results

### 3.1 Analyses of clinical characteristics between NAION group and control group

The current study comprised 162 individuals in total, including 61 individuals in NAION group (mean age 63.2 ± 9.7 years and 65% male) and 101 individuals in control group (mean age 61.7 ± 8.5 years and 62% male). Table 1 shows the demographic and medical characteristics of all subjects. Hypertension was most frequently observed in all clinical characteristics, with dyslipidemia and carotid artery stenosis following it. There were no statistically significant differences observed in age, gender, and other clinical characteristics between the control group and the NAION group.

**Table 1:** Clinical characteristics of NAION patients compared to controls.

Characteristics	NAION (*n* = 61)	Control (*n* = 101)	*p*-value
Age, mean, y	63.2 ± 9.7	61.7 ± 8.5	0.318
Sex, male, *N* (%)	41 (65)	63 (62)	0.729
Past medical history, *N* (%)			
Carotid artery stenosis	23 (36)	37 (37)	0.989
History of stroke/TIA	6 (10)	16 (15)	0.358
Cardiovascular disease	11 (17)	19 (18)	0.991
Diabetes mellitus	15 (24)	26 (26)	0.926
Hypertension	40 (63)	66 (65)	0.941
Dyslipidemia	33 (52)	51 (50)	0.941
Smoking	22 (35)	30 (30)	0.598
Alcohol consumption	18 (29)	33 (33)	0.705
Obesity	18 (29)	25 (25)	0.720

### 3.2 Comparison of distribution of SVDs between NAION group and control group

NAION group had a significantly higher frequency in presence of SVDs compared to those in control group (82 and 53%, *p* < 0.001). Among subgroups of SVDs, individuals with NAION were shown to have greater frequencies of WMH and PVS compared to control group, but lacunar infarction (35 and 26%, *p* = 0.281) and CMB (12 and 8.9%, *p* = 0.609) were not different significantly. Moreover, the medians of WMH score in NAION group and control group were 2 with an interquartile range (IQR) of 1–3 and 0 with an IQR of 0–2, respectively. The medians of CSVD score in NAION group and control group were 1 (IQR, 0–2) and 0 (IQR, 0–1). Individuals in the NAION group had substantially higher WMH and CSVD scores compared to the control group (*p* < 0.001). Table 2 presents the results of brain MRI scans for two groups.

**Table 2:** Brain MRI scan comparison between NAION patients and controls.

Characteristics *N* (%)	NAION (*n* = 63)	Control (*n* = 101)	*p*-value
Presence of CSVDs	52 (82)	54 (53)	<0.001
Lacunar infarction	22 (35)	26 (26)	0.281
CMB	8 (12)	9 (8.9)	0.609
PVS score	0 [0–2]	0 [0–0]	<0.001
0	35 (55)	88 (87)	
1	9 (14)	7 (6.9)	
2	9 (14)	5 (5.0)	
3	7 (11)	1 (1.0)	
4	3 (4.8)	0 (0.0)	
WMH score	2 [1–3]	0 [0–2]	<0.001
0	14 (22)	53 (53)	
1	5 (7.9)	9 (8.9)	
2	13 (21)	22 (22)	
3	17 (27)	10 (9.9)	
4	14 (22)	7 (6.9)	
CSVD score	1 [0–2]	0 [0–1]	<0.001
0	16 (25)	55 (54)	
1	17 (27)	24 (24)	
2	21 (33)	19 (19)	
3	6 (9.5)	2 (2.0)	
4	3 (4.8)	1 (1.0)	

### 3.3 Predictors of presence of SVDs and increased SVDs severity

The association between the SVDs and clinical risk factors was analyzed with the multiple logistic analysis and shown in Table 3. The models with minimal Akaike’s Information Criterion were selected. After adjusting for the effects of potential confounders, the OR of SVDs was 4.11 times greater in NAION group compared to the control group (95% CI: 1.93–8.79, *p* < 0.001) (Table 3). An ordinal logistic regression was performed to investigate the relevant clinical characteristics of WMH score, PVS score and CSVD score. Patients in NAION group had 3.08, 5.66 and 2.90-times higher risk than in control group, at each point of WMH score (95%CI: 1.43–6.79, *p* = 0.003), PVS score (95%CI: 2.31–14.9, *p* < 0.001) and CSVD score (95%CI: 1.32–6.51, *p* = 0.005) respectively. Moreover, dyslipidemia appeared to be the risk factor of SVDs (OR = 2.31, 95%CI: 1.20–4.44, *p* = 0.008) and WMH score (OR = 2.22, 95%CI: 1.07–4.70, *p* = 0.018). Furthermore, patients with history of stroke or TIA were more likely to develop SVDs (OR = 4.00, 95%CI: 1.13–14.17, *p* = 0.004) and have higher marks in PVS (OR = 3.54, 95%CI: 1.05–11.8, *p* = 0.021) and total CSVDs burden (OR = 4.67, 95%CI: 1.58–14.2, *p* = 0.003).

**Table 3:** Results of multiple regression analysis of NAION and clinical risk factors affecting SVD.

Variables	Presence of CSVDs OR (95% CI) *p*-value		WMH score OR (95% CI) *p*-value		PVS score OR (95% CI) *p*-value		CSVD score OR (95% CI) *p*-value	
NAION	4.11 (1.93–8.79)	<0.001	3.08 (1.43–6.79)	0.003	5.66 (2.31–14.9)	<0.001	2.90 (1.32–6.51)	0.005
Age (y)	1.10 (1.05–1.15)	<0.001	1.07 (1.03–1.12)	0.002	0.98 (0.93–1.03)	0.243	1.03 (0.97–1.07)	0.201
Sex (male)	1.15 (0.59–2.25)	0.679	0.75 (0.31–1.86)	0.264	1.16 (0.43–3.16)	0.380	0.61 (0.25–1.50)	0.143
Carotid artery stenosis	1.03 (0.53–1.99)	0.941	0.51 (0.24–1.09)	0.044	0.72 (0.29–1.72)	0.232	0.51 (0.23–1.13)	0.051
History of stroke/TIA	4.00 (1.13–14.17)	0.004	2.42 (0.87–6.93)	0.048	3.54 (1.05–11.8)	0.021	4.67 (1.58–14.2)	0.003
Cardiovascular disease	0.66 (0.30–1.48)	0.093	0.62 (0.20–1.89)	0.199	0.64 (0.15–2.34)	0.260	0.56 (0.18–1.68)	0.154
Diabetes	1.44 (0.67–3.10)	0.347	0.78 (0.32–1.90)	0.295	1.25 (0.45–3.39)	0.335	1.42 (0.58–3.43)	0.220
Hypertension	1.68 (0.86–3.25)	0.131	1.75 (0.80–3.85)	0.082	2.27 (0.90–6.21)	0.048	1.94 (0.84–4.58)	0.063
Dyslipidemia	2.31 (1.20–4.44)	0.008	2.22 (1.07–4.70)	0.018	1.70 (0.72–4.17)	0.119	1.68 (0.80–3.60)	0.088
Smoking	0.73 (0.37–1.44)	0.022	1.09 (0.38–3.06)	0.437	1.26 (0.35–4.74)	0.364	0.76 (0.27–2.15)	0.303
Alcohol consumption	1.47 (0.72–3.00)	0.027	1.04 (0.39–2.74)	0.467	1.05 (0.28–3.64)	0.470	1.15 (0.43–3.16)	0.390
Obesity	0.46 (0.23–0.94)	0.136	0.93 (0.37–2.31)	0.435	0.73 (0.24–2.02)	0.280	0.87 (0.34–2.18)	0.385

## 4 Discussion

This study evaluated the brain MRI and several vascular risk factors of individuals with NAION compared to a control group matched for sex and age. Moreover, we evaluated total burden CSVD score and investigated the association between NAION and severity of SVDs. This work is the first retrospective case–control study to utilize the total CSVD score for a quantitative assessment of the correlation between NAION and SVDs. Our findings revealed that there was an association between NAION and SVDs after adjusting for other risk factors. Furthermore, NAION patients tended to have an increased risk of higher total CSVD score. Therefore, our results suggest that NAION can be considered as an independent risk factor in predicting the development of SVDs.

Previous studies have reported SVDs was associated with NAION (9). Kim et al. retrospectively reviewed brain MRI results of 63 individuals with NAION and compared them with controls with no neurological conditions. After adjusting for other risk factors, the NAION group was found to be higher proportion in presence of SVDs than the control group. The earlier findings agree with results of the present study. Although previous studies investigated SVDs findings consisted of three separate subtypes (WMH, CMB, and lacunar infarctions), the total severity of SVDs should be considered. Our research indicated that patients with NAION have an increase in the overall burden of SVDs, as determined by the total CSVD score. This inspired us to pay more attention to patients with NAION in clinical practice to prevent severe SVDs. Since SVDs cause cognitive impairment and severe SVDs lead to stroke and dementia, we advise referring individuals who have NAION for brain MRIs and controlling risk factors related to SVDs.

Our study indicated that brain MRI of NAION patients exhibited higher-grade WMH and PVS lesions. Considering this correlation, it is reasonable to assume that NAION share comparable pathologies with WMH and PVS. Impaired autoregulatory mechanisms cause hypoperfusion rather than arteriosclerotic vessel changes. Hayreh found that hypoperfusion in micro-circulation around optic nerve may cause long-term damage optic nerve head circulation, especially in individuals with systematic hypertension in whom blood pressure fluctuates greatly at night leading to impaired autoregulatory mechanisms in optic disc circulation (2). Similarly, previous studies investigated hypoxia, alternative microglial cells and blood-barrier dysfunctions played important roles in the pathogenesis of WMH. The earlier study in Alzheimer’s disease, vascular permeability was changed and cerebral blood flow autoregulation was disrupted due to Wallerian degeneration or deleterious effects of amyloid (15, 16). Additionally, inflammation contributes to the development of both NAION and PVS. Previous study indicated that patients with systemic lupus erythematous had significantly higher PVS frequency on brain MRI than healthy age-matched controls (17). Wang et al. found that the accumulation of macrophage cells at the vitreoretinal interface in eyes with acute NAION is significantly increased, particularly in areas that correlated to visual field defects (18).

Moreover, our study found that lacunar infarction was not related to NAION. This suggest that NAION is a different pathophysiological process from lacunar infarction. The mechanism of lacunar infarction is acute occlusion of a small vessel, which subsequently results in tissue necrosis and ischemia (11). However, in the fluorescein fundus angiography study, Hayreh et al. found more than 1,000 eyes with acute, classical NAION, no such occlusion was ever seen in the posterior ciliary artery due to thrombosis (2). In addition, since there are similar risk factors for both cerebral ischemic stroke and NAION, previous studies evaluated the correlation of NAION and cerebral ischemic stroke. However, due to overlapping comorbidities and vascular risks, the direct association between NAION and cerebral ischemic stroke have not been proved (19, 20). Thus, we presume that NAION is not a thromboembolic disorder.

Furthermore, we found that the incidence of dyslipidemia was prone to SVDs and it was related to a higher risk of worse WMH. Previous systematic review and meta-analysis study demonstrated dyslipidemia and raised lipoprotein were significantly related to a greater risk of NAION compared to hypertension and diabetes (21), thus suggesting that dyslipidemia may be a key factor in pathogenic process of NAION. On the other hand, Cheng et al. found that cognitive alternation with low total SVDs burden was not significantly related to vascular risk factor, but statin therapy for dyslipidemia may delay the progression of advanced WMH and reduce conversion to dementia (22). This association provides evidence to support that dyslipidemia may contribute to neurodegeneration in pathogenesis of WMH. The above findings support this study’s results. We speculate that same mechanisms are responsible for pathogenic alteration in patients with NAION and support further interventional study in neurodegeneration.

There are several limitations in this retrospective research. First, the number of NAION patients that could be collected at a single hospital was a limited sample size, especially in multivariate regression analysis. Second, owing to a selection bias, patients with ischemic stroke or TIA were not excluded. Patients in our study, who are intentionally visit medical imaging department in the hospital, are more likely to undergo neurological symptoms. Third, data on other factors, including eating patterns and life styles, which may be potential confounding factors that could affect SVDs and NAION, are not available in our medical records. Finally, due to the limitation of retrospective analysis of this study, it is not yet possible to evaluate the temporal relationship between NAION and the onset of CSVDs. The efficacy of NAION in predicting CSVDs requires further assessment through more prospective studies in healthy individuals.

## 5 Conclusion

This study demonstrated that NAION had a predictive impact on development of SVDs. A significant correlation was found between the incidence of NAION and the rise in the total CSVD score. We also found the evidence demonstrating that NAION was concurrent with increased WMH score and PVS score. Therefore, we speculate that NAION and SVDs share a similar pathogenic mechanism and suggest that patients with NAION should be paid more attention on SVDs development. Further large-scale prospective multicenter studies are required to thoroughly assess the correlation between NAION and SVDs.

## Data Availability

The raw data supporting the conclusions of this article will be made available by the authors, without undue reservation.
